# Care trajectories around a first dementia diagnosis in patients with serious mental illness

**DOI:** 10.1111/ggi.14889

**Published:** 2024-05-06

**Authors:** Isabelle Dufour, Sébastien Brodeur, Josiane Courteau, Marc‐André Roy, Alain Vanasse, Amélie Quesnel‐Vallee, Isabelle Vedel

**Affiliations:** ^1^ Nursing School Université de Sherbrooke Sherbrooke Québec Canada; ^2^ Research Center on Aging Université de Sherbrooke Sherbrooke Québec Canada; ^3^ Department of Psychiatry and Neurosciences Université Laval Québec City Québec Canada; ^4^ PRIMUS Research group, CHUS Research center Sherbrooke Québec Canada; ^5^ Department of Psychiatry and Neurosciences Université Laval Québec Québec Canada; ^6^ CERVO Brain Research Group Québec Québec Canada; ^7^ Department of Family and Emergency Medicine Université de Sherbrooke Sherbrooke Québec Canada; ^8^ Department of Sociology, Faculty of Arts McGill University Montreal Québec Canada; ^9^ Department of Epidemiology, Biostatistics, and Occupational Health McGill University Montreal Québec Canada; ^10^ McGill Observatory on Health and Social Services Reforms Montreal Québec Canada; ^11^ Department of Family Medicine, Faculty of Medicine and Health Sciences McGill University Montréal Québec Canada; ^12^ Lady Davis Institute for Medical Research Jewish General Hospital Montréal Québec Canada

**Keywords:** bipolar disorder, care trajectory, dementia, healthcare services, schizophrenia

## Abstract

**Aim:**

To develop a typology of care trajectories (CTs) 1 year before and after a first dementia diagnosis in individuals aged ≥65 years, with prevalent schizophrenia or bipolar disorder.

**Methods:**

This was a longitudinal, retrospective cohort study using health administrative data (1996–2016) from Quebec (Canada). We selected patients aged ≥65 years with an incident diagnosis of dementia between 1 January 2014 and 31 December 2016, and a diagnosis of schizophrenia and/or or bipolar disorder. A CT typology was generated by a multidimensional state sequence analysis based on the “6 W” model of CTs. Three dimensions were considered: the care setting (“where”), the reason for consultation (“why”) and the specialty of care providers (“which”).

**Results:**

In total, 3868 patients were categorized into seven distinct types of CTs, with varying patterns of healthcare use and comorbidities. Healthcare use differed in terms of intensity, but also in its distribution around the diagnosis. For instance, whereas one group showed low healthcare use, healthcare use abruptly increased or decreased after the diagnosis in other groups, or was equally distributed. Other significant differences between CTs included mortality rates and use of long‐term care after the diagnosis. Most patients (67%) received their first dementia diagnosis during hospitalization.

**Conclusions:**

Our innovative approach provides a unique insight into the complex healthcare patterns of people living with serious mental illness and dementia, and provides an avenue to support data‐driven decision‐making by highlighting fragility areas in allocating care resources. **Geriatr Gerontol Int 2024; 24: 577–586**.

## Introduction

People living with dementia (PLWD) often experience severe and persistent disabilities, participation restrictions, high formal and informal care needs, and higher risks of adverse outcomes (e.g. hospitalization, emergency department visits and institutionalization).^1^, ^2^ Furthermore, specific subgroups of PLWD show particular complexity because of their inherent characteristics and previous patterns of healthcare use,^3^ notably individuals with serious mental illness (SMI), such as bipolar disorder (BPD) or schizophrenia (SCZ), the most common SMI types.^4^, ^5^

In all ages, BPD/SCZ are associated with higher rates of comorbidity, premature frailty and mortality.^6^, ^7^ The prevalence of dementia increases rapidly with age in individuals with SMI,^8^, ^9^ with this population showing over twice the risk for dementia, whereas approximately 10% of PLWD also have SMI.^8^, ^9^, ^10^, ^11^, ^12^ However, the identification, diagnosis and treatment of dementia are further complicated in individuals with BPD/SCZ; BPD/SCZ being themselves characterized by cognitive impairments, distinguishing those from newly developed dementia can be challenging.^8^, ^13^, ^14^ Furthermore, inequities regarding access to preventive and primary care are also reported, as individuals living with BPD/SCZ tend to receive fewer and lower quality services, and experience more care transitions.^15^, ^16^

Strategic knowledge of the complex dynamics underlying healthcare use in PLWD presenting with BPD/SCZ could be gained by describing care trajectory (CT), defined as the healthcare use pattern across time.^17^ CT might significantly impact patient morbidity, mortality and quality of life, in addition to individual and clinical features.^17^, ^18^ This understanding is crucial to identify fragility areas and improve patient outcomes, yet literature on CT and healthcare use patterns regarding this specific population is scarce.^19^, ^20^ Nevertheless, two studies showed higher rates of hospitalizations, psychiatric visits, emergency department visits, and institutionalization in patients with PLWD and SMI compared with those with dementia alone, increasing their risk of adverse outcomes and potentially evitable care transitions.^8^, ^9^

Building on these findings and in response to significant knowledge gaps, we sought to examine longitudinal and multidimensional CT (according to healthcare settings, types of professionals consulted and reason for consulting) while accounting for population heterogeneity.^10^ To do so, we used a modified multidimensional approach of state sequence analysis (SSA), an innovative statistical method enabling complex longitudinal patterns visualization.^21^ Specifically, we aimed to: (1) propose a multidimensional typology of CTs before and after a first dementia diagnosis in individuals with BPD/SCZ; and (2) describe and compare their characteristics by CT typology.

## Methods

This was a retrospective cohort study from the province of Quebec, Canada. Our approach of CT builds on the ‘6 W’ multidimensional model,^17^ which conceptualizes patterns of care use into six dimensions: patients (*Who*), in response to their healthcare conditions and needs (*Why*), will consult different care providers (*Which*) in different settings (*Where*), where they will receive tests and treatments (*What*) at specific times (*When*).

### Design and data sources

The present study was based on a health administrative database composed of individuals with SMI (such as BPD/SCZ, and other types of psychosis; *n* = 380 124 patients, 1996–2016). We acquired the database from the provincial health insurance board (Régie de l'assurance maladie du Québec), which manages universal health insurance for Quebec residents.^22^

The Régie de l'assurance maladie du Québec gives access to: (1) patient demographic information (e.g. date of birth and death, geographical location of residency); (2) medical services register (information from physicians' claims for services provided in outpatient clinics, emergency and primary care clinics, including date of service and diagnosis coded with the International Classification of Diseases 9 [ICD‐9]); (3) provincial public drug insurance plan eligibility (e.g. insurance status); (4) pharmaceutical services (data on each drug claimed in a pharmacy); (5) hospital discharge register (information on hospitalizations' date, length of stay, primary and secondary diagnoses (ICD‐9 before April 2006; ICD‐10 thereafter)); and (6) I‐CLSC database (available since April 2012, and containing a wide range of primary and home care services provided by local community service centers [CLSC] by nurses, social workers etc.). Patient data from these registers were linked using a unique encrypted identifier.

### Studied population

**Figure 1:**
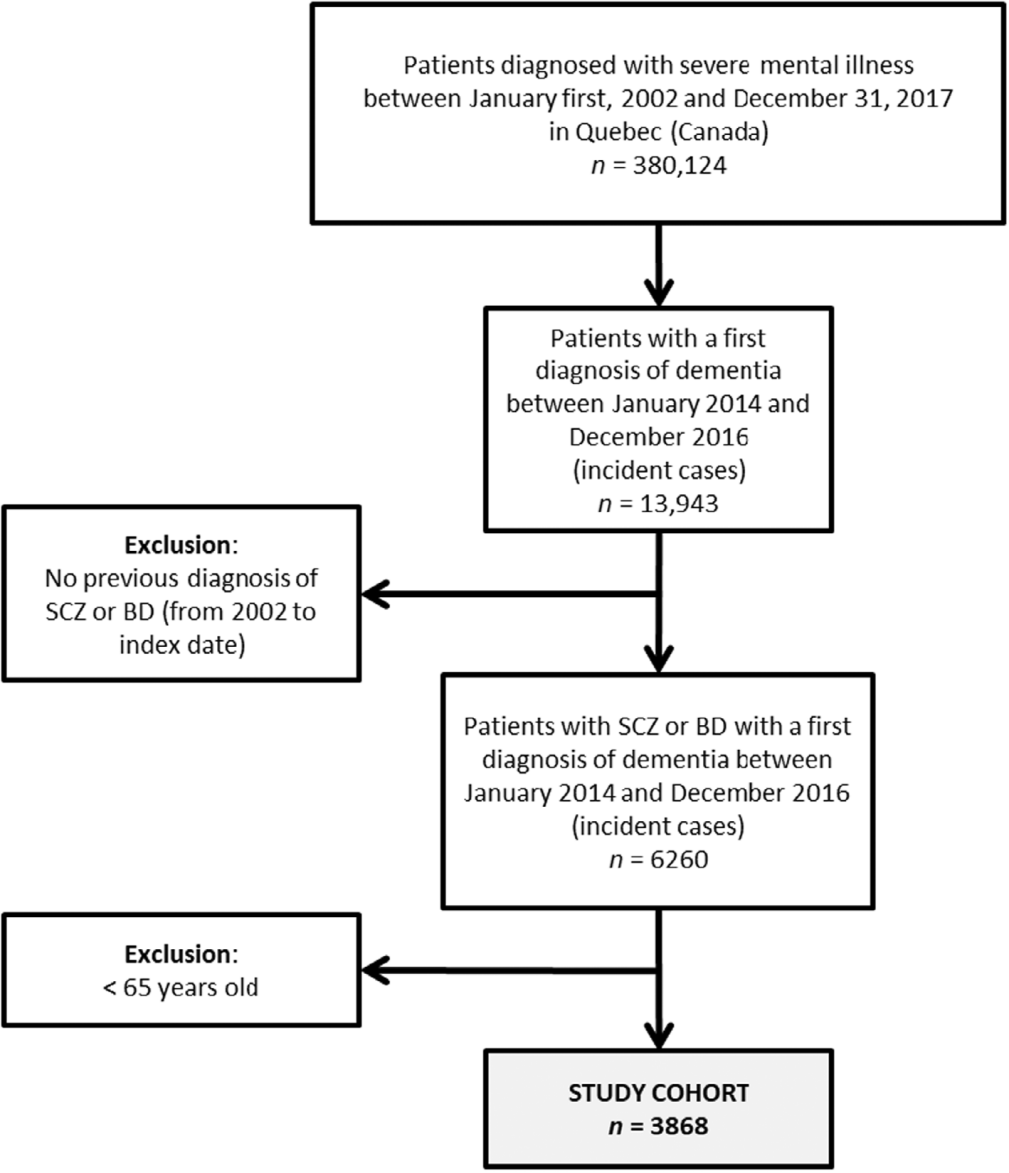
Study cohort flow diagram. SCZ, schizophrenia; BD, bipolar disorder.

The study cohort included all patients aged ≥65 years with a first diagnosis of dementia (incident cases) registered between 1 January 2014 and 31 December 2016 (index date: the date of the dementia diagnosis) and a diagnosis of BPD/SCZ (Fig. 1). Dementia cases were defined as patients receiving a dementia diagnosis (ICD‐9: 290, 294.1, 331.0, 331.2; ICD‐10: F00‐F03, F05.1, G30, G31.1) during a hospitalization or at least two diagnoses of dementia (ICD‐9: 290, 294.1, 331.0, 331.2) during two different medical visits within 2 years.^23^ To exclude prevalent cases, we removed patients with a previous dementia diagnosis between January 2002 and the index date. Only patients with a prior diagnosis of BPD/SCZ (ICD‐9: 295, 296; ICD‐10: F20, F21, F23.2, F25, F30, F31) were included.

## Variables

### Demographics and clinical characteristics

Characteristics of patients (dimension *Who*) first included age and sex. In the 2 years before the index date, we considered: (1) a comorbidity index,^23^ from which dementia and BPD/SCZ were excluded; (2) physical conditions; and (3) the number of drugs. At the index date, we considered: (1) the admissibility status of the public prescription drug insurance plan (as a proxy measure of low‐income/unemployment status) and included four categories: not admissible (people with a private drug insurance plan); admissible and age ≥65 years with guaranteed income supplement; admissible and being a recipient of last resort financial assistance; or regular recipient; (2) the residential neighborhood's material and social deprivation indices,^24^ based on geographic area (quintile 1 – least deprived – to quintile 5 – most deprived); (3) residential neighborhood characteristics (metropolitan area: ≥100 000 inhabitants; small town: 10 000–100 000 inhabitants; rural: <10 000 inhabitants). In the year after the index date, we considered death, home care and long‐term care admission.^25^

### Statistical analysis

We used a multidimensional SSA approach to characterize CTs and define homogeneous groups.^26^ CTs were measured over 2 years (1 year before and after the index date – the date of the dementia diagnosis), and “weeks” was chosen as the time units (104 weeks). We defined the following dimension‐specific states (each following a priority order) for each week (*When*): (1) The *Where* dimension: hospital, emergency department, outpatient clinic, primary care or private clinic, local community service center (CLSC – home care services) and no such healthcare use; (2) The *Why* dimension (reasons for healthcare use: dementia, dementia [interventions/consultations in CLSC settings]), mental disorders other than dementia, non‐mental disorders and no such healthcare use; and (3) The *Which* dimension: geriatricians or neurologists, psychiatrists, other MD specialists, general practitioners, nurses or psychologists or social workers in CLSC, and other professionals in CLSC (e.g. beneficiary attendants), and no such healthcare use.

Then, for each patient, we created three sequences of healthcare use, one per dimension (*Where*, *Why*, *Which*). A distance matrix containing the distance (proximity) between each pair of patients' CTs was calculated for each dimension.^26^ A pooled distance matrix between CT sequences was calculated by summing the three dimension‐specific distances, and then a hierarchical cluster analysis was used to classify patients with similar unique CTs.^27^ We based the optimal number of CT types on statistical criteria (dendrogram and inertia curve), parsimony, interpretability and clinical judgment. The final solution is the result of a consensus between five clinicians.

We used two visual representations to interpret the types of CTs: (1) state distribution plots, showing the distribution of states for each time unit point; and (2) the mean number of days in each state for each dimension. Once each patient was classified in a specific cluster (with similar CTs), covariables between groups were compared using Kruskal–Wallis and χ^2^‐tests. The SSA was carried out using the TraMineR package in R (The R Foundation for Statistical Computing, Vienna, Austria).^28^ All other analyses were carried out using SAS 9.4 (SAS Institute, Cary, NC, USA).

## Results

**Figure 2:**
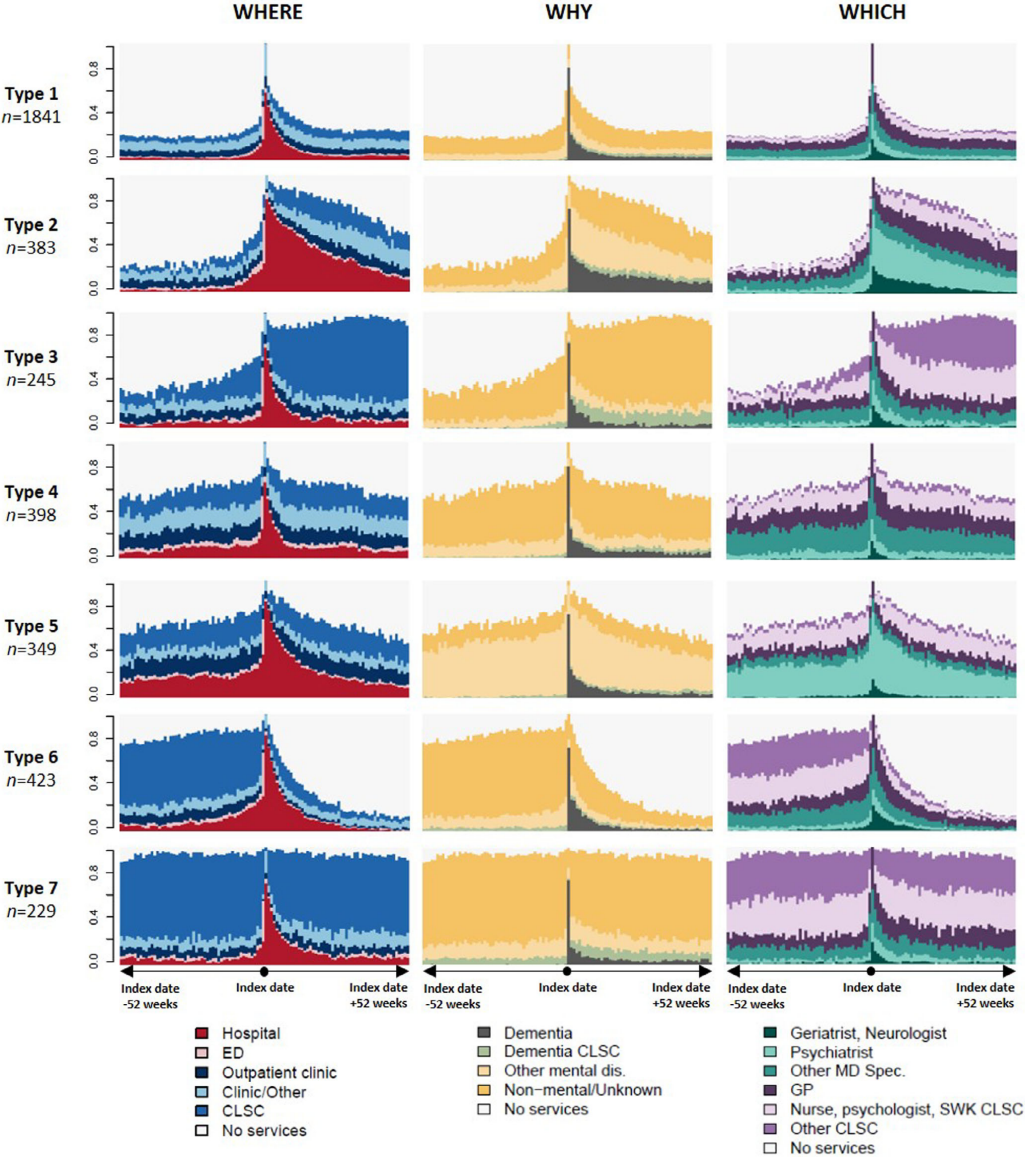
State distribution plots of the typology of care trajectories by dimension (Where, Why and Which). State distribution plots show the distribution of states (proportion) for each week of follow up (52 weeks before and 52 weeks after a first dementia diagnosis). ED, emergency department; CLSC, local community service centers; GP, general practitioner; SWK, social worker.

**Figure 3:**
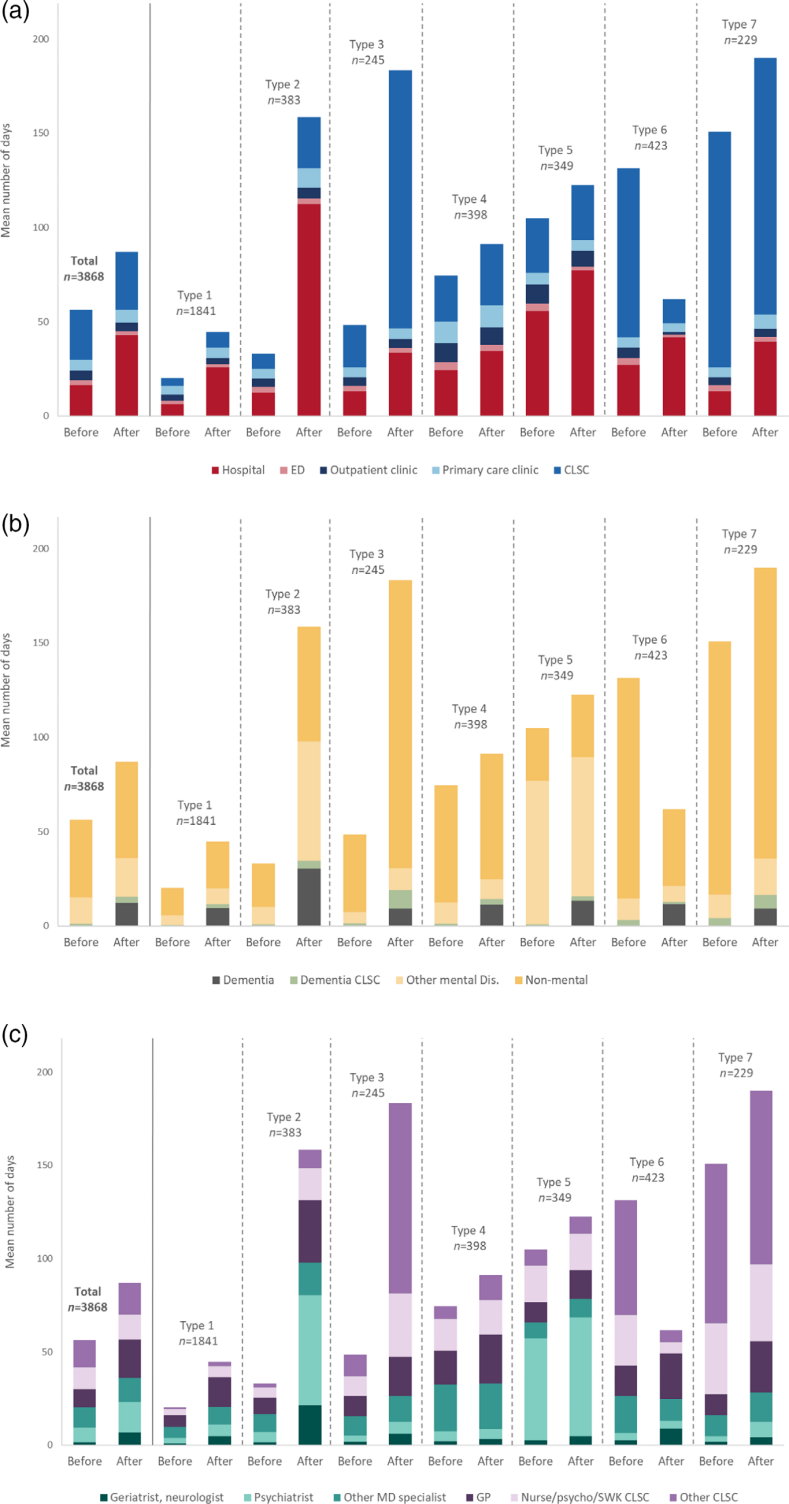
Mean number of days of healthcare use according to the (a) care setting (where), (b) reason for consultation (why), and (c) care provider (which) 1 year before and 1 year after the diagnosis of dementia. ED, emergency department; CLSC, local community service centers; GP, general practitioner; SWK, social worker.

The study cohort included 3868 individuals presenting with BPD/SCZ who received a first dementia diagnosis between January 2014 and December 2016. Overall, individuals had a mean age of 77 years (IQR 71–73 years), with a majority of women (63.9%) and predominantly residing in metropolitan areas (72.8%). Our SSA approach showed seven distinct CT types (or groups; see Figs 2 and 3).

Group 1 (*n* = 1841, 47.6%) was the most common CT type, and could be described as low overall healthcare users (Figs 2 and 3). They had the lowest comorbidity, medication intake and mental health consultations. Their mortality and long‐term care utilization rates were approximately 15% each (Table 1). Group 2 (*n* = 383, 9.9%) similarly showed low healthcare use, but solely before the dementia diagnosis (Fig. 2). Compared with group 1, group 2 also showed higher rates of hospitalization days, and consultations with psychiatrists, geriatrists and neurologists (Fig. 3). Furthermore, 26.9% were transferred to long‐term care (Table 1). Group 3 (*n* = 245, 6.3%) showed a gradual increase in non‐mental healthcare use before the dementia diagnosis (Fig. 2). In the subsequent year, they showed the greatest utilization of home care (*n* = 237, 96.7%), with minimal reliance on long‐term care (<5%) and a low mortality rate (<3%; see Table 1). Group 4 (*n* = 398, 10.3%) consisted of patients with higher comorbidity (Table 1), who maintained consistent healthcare use before and after dementia diagnosis (Fig. 2). Over the 2‐year span, they were more frequent users of primary care clinics (Figs 2 and 3). Group 5 (*n* = 349, 9%) comprised younger individuals (Table 1), with the highest intensity of non‐dementia mental health‐related and psychiatric consultations throughout the study period (Figs. 2 and 3).

**Table 1:** Characteristics of the study cohort by the typology of care trajectories.

Characteristics	Total	Type 1	Type 2	Type 3	Type 4	Type 5	Type 6	Type 7	*P*‐value
	*n* = 3868	*n* = 1841 (47.6%)	*n* = 383 (9.9%)	*n* = 245 (6.3%)	*n* = 398 (10.3%)	*n* = 349 (9.0%)	*n* = 423 (10.9%)	*n* = 229 (5.9%)	
Age (years), median (IQR)^†^	77 (71–83)	77 (71–83)	75 (70–81)	78 (72–83)	78 (71–83)	73 (68–78)	80 (74–86)	81 (69–81)	<0.0001
Age group, *n* (%)^†^									<0.0001
65–74 years	1520 (39.3)	720 (39.1)	178 (46.5)	83 (33.9)	150 (37.7)	214 (61.3)	115 (27.2)	60 (26.2)	
75–84 years	1569 (40.6)	765 (41.6)	147 (38.4)	120 (49.0)	163 (41.0)	107 (30.7)	170 (40.2)	97 (42.4)	
≥85 years	779 (20.1)	356 (19.3)	58 (15.1)	42 (17.1)	85 (21.4)	28 (8.0)	138 (32.6)	72 (31.4)	
Sex, *n* (%)									0.0267
F	2471 (63.9)	1170 (63.6)	225 (58.8)	160 (65.3)	258 (64.8)	217 (62.2)	273 (64.5)	168 (73.4)	
M	1397 (36.1)	671 (36.4)	158 (41.2)	85 (34.7)	140 (35.2)	132 (37.8)	150 (35.5)	61 (26.6)	
PPDIP status, *n* (%)^†^									<0.0001
Not admissible	370 (9.6)	205 (11.1)	23 (6.0)	7 (2.9)	58 (14.6)	31 (8.9)	41 (9.7)	5 (2.2)	
Admissible – regular	1254 (32.4)	650 (35.3)	130 (33.9)	80 (32.6)	127 (31.9)	73 (20.9)	124 (29.3)	70 (30.6)	
Admissible – LRFA/GIS	2244 (58.0)	986 (53.6)	230 (60.0)	158 (64.5)	213 (53.5)	245 (70.0)	258 (61.0)	154 (67.2)	
Material deprivation quintiles, *n* (%)^†^, ^‡^									0.5038
1 (less deprived)	555 (16.6)	272 (17.3)	54 (16.1)	34 (15.5)	59 (17.3)	51 (16.7)	63 (17.3)	22 (11.1)	
2–4	117 (59.6)	942 (60.0)	206 (61.3)	125 (57.1)	203 (59.5)	185 (60.7)	208 (57.1)	117 (59.1)	
5 (most deprived)	792 (23.8)	356 (22.7)	76 (22.6)	60 (27.4)	79 (23.2)	69 (22.6)	93 (25.6)	59 (29.8)	
Social deprivation quintiles, *n* (%)^†^, ^§^									0.5658
1 (less deprived)	406 (12.2)	194 (12.4)	34 (10.1)	26 (11.9)	40 (11.7)	43 (14.1)	48 (13.2)	21 (10.6)	
2–4	1808 (54.2)	873 (55.6)	177 (52.7)	127 (58.0)	182 (53.4)	160 (52.5)	181 (49.7)	108 (54.6)	
5 (most deprived)	1119 (33.6)	503 (32.0)	125 (37.2)	66 (30.1)	119 (34.9)	102 (33.4)	135 (37.1)	69 (34.8)	
Rurality, *n* (%)^†^, ^‡^									0.0105
Metropolitan	2676 (72.8)	1286 (73.5)	296 (79.1)	165 (68.5)	282 (72.5)	237 (70.5)	260 (72.2)	150 (66.1)	
Small town	429 (11.7)	211 (12.1)	31 (8.3)	31 (12.9)	52 (13.4)	32 (9.5)	37 (10.3)	35 (15.4)	
Rural	572 (15.6)	253 (14.5)	47 (12.6)	45 (18.7)	55 (14.1)	67 (19.9)	63 (17.5)	42 (18.5)	
Comorbidity index, *n* (%)^¶^									<0.0001
0	1250 (32.3)	774 (42.0)	130 (33.9)	67 (27.4)	55 (13.8)	81 (23.2)	86 (20.3)	57 (24.9)	
1–2	898 (23.2)	461 (25.0)	88 (23.0)	61 (24.9)	95 (23.9)	74 (21.2)	70 (16.6)	49 (21.4)	
3–5	670 (17.3)	281 (15.3)	72 (18.8)	47 (19.2)	83 (20.8)	68 (19.5)	74 (17.5)	45 (19.6)	
≥6	1050 (27.2)	325 (17.6)	93 (24.3)	70 (28.6)	165 (41.5)	126 (36.1)	193 (45.6)	78 (34.1)	
Physical condition, *n* (%)^¶^									
Cardiac condition	1006 (26.0)	351 (19.1)	94 (24.5)	65 (26.5)	152 (38.2)	92 (26.4)	176 (41.6)	76 (33.2)	<0.0001
COPD	948 (24.5)	331 (18.0)	96 (25.1)	61 (24.9)	132 (33.2)	99 (28.4)	156 (36.9)	73 (31.9)	<0.0001
Cancer	648 (16.8)	264 (14.3)	59 (15.4)	46 (18.8)	99 (24.9)	51 (14.6)	97 (22.9)	32 (14.0)	<0.0001
First dementia diagnosed during hospitalization, *n* (%)	2592 (67.0)	1061 (57.6)	305 (79.6)	171 (69.8)	260 (65.3)	288 (82.5)	348 (82.3)	159 (69.4)	<0.0001
Death, *n* (%)^††^	600 (15.5)	278 (15.1)	27 (7.0)	< 3%	47 (11.8)	34 (9.7)	205 (48.5)	< 3%	<0.0001
Long‐term care, *n* (%)^††^	716 (18.5)	282 (15.3)	103 (26.9)	12 (4.9)	70 (17.6)	67 (19.2)	157 (37.1)	25 (10.9)	<0.0001
Home care (nurse, psychosocial, SWK), *n* (%)^††^	2844 (73.5)	1097 (59.6)	297 (77.6)	237 (96.7)	329 (82.7)	267 (76.5)	388 (91.7)	229 (100)	<0.0001
No. Rx, median (IQR)^¶^	13 (8–18)	10 (6–15)	12 (7–16)	14 (9–19)	16 (11–23)	14 (10–19)	16 (11–21)	17 (12–22)	<0.0001

^†^ Variable considered at the index date.

^‡^ Missing values; *n* = 535.

^§^ Missing values; *n* = 191.

^¶^ Variable calculated in the 2 years preceding the index date.

^††^ Variable considered in the year following the index date.

Abbreviations: COPD, chronic obstructive pulmonary disease; GIS, guaranteed income supplement; IQR, interquartile range; LRFA, last‐resort financial assistance; PPDIP, provincial public drug insurance plan; SWK, social worker.

For several groups (groups, 3, 4, 6 and 7), consultations were mostly driven by physical needs (i.e. non‐mental causes), although with different patterns before and after the diagnosis: more frequent consultations for non‐mental causes were found in the year preceding dementia diagnosis for group 6, in the year after the diagnosis for group 3, and in both years for groups 4 and 7 (Figs 2 and 3). Groups 3, 6 (*n* = 423, 10.9%) and 7 (*n* = 229, 5.9%) also included more support services from CLSC, including home‐care services, in the year preceding dementia diagnosis for group 6, the year following the diagnosis for group 3 and both years for group 7 (Figs 2 and 3). In the year after the diagnosis, we observed high rates of long‐term care (37.1%) and death (48.5%) in group 6 (Table 1), along with a rapid decrease in healthcare use (Fig. 2), whereas these rates were, respectively, 10.9% and <3% for group 7 (Table 1). Group 6, which includes aged individuals with more comorbidities (Table 1), is also characterized by a high utilization of home care services (91.7%) that concurs with the marked decrease in healthcare use after the diagnosis (Fig. 2). In group 7, the high intensity of services received before the dementia diagnosis concurs with high physical needs, a pattern maintained after the diagnosis (Figs 2 and 3).

## Discussion

To our knowledge, this is the first study aiming to propose a typology of CTs before and after a first dementia diagnosis in individuals with BPD/SCZ. Using an innovative multidimensional SSA approach, we identified seven distinct patterns of healthcare use, offering a comprehensive perspective of the phenomenon and showing significant variation within our population.

The low healthcare service utilization, particularly related to mental disorders, either throughout the study period (group 1) or mainly after the dementia diagnosis (group 6), aligns with approximately 50% of the broader PLWD population who show a similar pattern.^11^ However, the extent to which the provided services align with individuals' healthcare needs remains to be determined, as mental illness is likely a source of distress and dysfunction,^29^ and SMI patients are known to receive less comprehensive care for physical illness.^30^, ^31^ Also, the fewer consultations related to mental disorders for groups 1, 2 and 3 before dementia diagnosis is more likely due to the under recognition of emerging dementia in SMI patients,^32^ rather than an abrupt dementia onset. Nevertheless, for groups 2 and 3, the diagnosis seems to act as a pivotal moment, prompting healthcare use intensity. This aligns with a previous study showing a significant increase in care transitions during the year of diagnosis, notably associated with poor health.^33^

For most groups, GP visits were infrequent, and 67% of patients were diagnosed during hospitalization. This contrasts with studies showing that approximately 25–45% of PLWD are diagnosed in hospital, with frequent GP contact.^7^, ^34^, ^35^ Given the concerns expressed by GPs in diagnosing dementia in the general population,^36^ it is quite plausible that these barriers appear even stronger for SMI patients, whose cases are more complex. Diagnosing dementia in hospital settings is not typically recommended, as it can notably extend stays and does not result in GP follow up or specialist referrals. An outpatient or memory clinic diagnosis provides comprehensive cognitive assessment, history consideration and patient‐centric care planning.^37^

Group 2 had more consultations with cognition specialists and higher rates of long‐term care placement after dementia diagnosis, possibly due to behavioral and psychological symptoms of dementia or lack of support.^38^, ^39^, ^40^ Behavioral and psychological symptoms of dementia affect approximately 60% of community‐dwelling PLWD,^41^, ^42^ intensify over time, and are associated with increased hospital stays, complications and earlier long‐term care admission.^41^, ^43^, ^44^ The frequent consultations for non‐mental causes in certain groups^3^, ^4^, ^6^, ^7^ might reflect the high co‐occurrence of physical comorbidities in SMI patients,^45^ and/or the altered health‐seeking behaviors in individuals with SMI, who prioritize physical symptoms over mental health‐related concerns.^46^

Group 5 consulted mainly for mental health reasons (other than dementia), both before and after the dementia diagnosis. This apparent contradiction with the general trend of reduced psychiatric symptoms with age^47^, ^48^ might be explained by the younger individuals present in this group. This observation aligns with existing literature, which suggests that younger individuals might continue to experience active psychiatric conditions despite the general trend of reduced psychiatric symptoms with age.^47^, ^48^ The results emphasize that group 5 is an exception rather than the norm regarding the frequency of interactions with the healthcare system for mental disorders.

Different trajectories regarding aging in place were also noticeable, group 6 notably experiencing more long‐term care placement, whereas individuals from group 7 remained at home with extensive CLSC services. Indeed, the proportion of PLWD in the community decreases with older age: ~33% of those aged <80 years and 42% of those aged ≥80 years reside in long‐term care homes.^49^ The higher rates of mortality and long‐term care placement in group 6, together with the marked decrease in healthcare utilization after the diagnosis and the more advanced age of individuals, suggest a late diagnosis, perhaps years after the real onset of dementia. The fact that diagnosing dementia in SMI patients is known to be challenging supports this hypothesis,^13^, ^14^ including the occurrence of cognitive decline within the course of SCZ itself.^50^, ^51^ Finally, in group 7, the high intensity of services received before the dementia diagnosis, and driven by their physical needs, might have contributed to this stability, despite specific information on care quality.^38^

The present study had several strengths. First, it used an exhaustive longitudinal dataset of individuals living with dementia and SMI; thus, the findings are generalizable to our population of interest. Our SSA approach enables the creation of homogeneous CTs types amidst complex populations. By adopting a multidimensional framework, we could incorporate 18 states organized into three dimensions, offering a comprehensive understanding of care patterns while enhancing interpretability. However, we could not consider important variables because of database limitations (e.g. individual variables, such as the severity of SMI or dementia, autonomy level, living situation and caregiver support). In addition, the composition of our trajectories could not consider the provision of community health services, or the support provided by community organizations or by professionals other than physicians, outside of the CLSC.

The patterns emerging from the present results help us reflect on existing practices to varying degrees. Canada and the USA have developed Alzheimer's disease plans, supporting dementia care mainly within primary care settings.^52^, ^53^ Nevertheless, these guidelines have overlooked the concurrent presence of SMI, underscoring the importance of ensuring preventive care access, robust identification and a comprehensive diagnostic process for individuals with SMI. In the same line, there is evidence that multiple barriers complexify access to physical health services for people with SMI, such as the division between physical and mental healthcare and stigmatization.^54^ Psychiatric illnesses most often stabilize over time; hence, patients newly acquiring cognitive symptoms should bring suspicion of dementia.^48^ Future research on CTs should be further oriented toward specific health outcomes, suboptimal transitions or early detection of dementia.

This is the first study to propose a complete portrait of healthcare use patterns before and after a first diagnosis of dementia in individuals with BPD/SCZ. Trajectory care approaches can help identify specific areas of improvement. The fact that nearly half our cohort (i.e. group 1) showed overall low healthcare utilization suggests that efforts should be directed toward other specific groups, although we cannot rule out the possibility of unmet needs among individuals from group 1. For instance, group 2, and group 5 to a lesser extent, showed considerable time spent in hospitals, especially after the diagnosis. Given that tertiary care settings are not optimal for PLWD care,^55^ solutions should aim to prevent hospitalizations as much as possible, while favoring ambulatory care. Our innovative approach provides a unique insight into the complex healthcare patterns of people living with SMI and dementia, and provides an avenue to support data‐driven decision‐making.

## Disclosure statement

The authors declare no conflict of interest.

## Ethics statement

This study was approved by the Research Ethics Board Committee of the Université de Sherbrooke and by the Commission d'accès à l'information of Quebec.

## Data Availability

The data that support the findings of this study are available on request from the corresponding author. The data are not publicly available due to privacy or ethical restrictions.
